# Emergence of SARS-CoV-2 Spike Mutations during Prolonged Infection in Immunocompromised Hosts

**DOI:** 10.1128/spectrum.00791-22

**Published:** 2022-05-11

**Authors:** Karrie K. K. Ko, Hatairat Yingtaweesittikul, Thuan Tong Tan, Limin Wijaya, Delphine Yanhong Cao, Sui Sin Goh, Nurdyana Binte Abdul Rahman, Kenneth X. L. Chan, Hui Ming Tay, James Heng Chiak Sim, Kian Sing Chan, Lynette L. E. Oon, Niranjan Nagarajan, Chayaporn Suphavilai

**Affiliations:** a Department of Microbiology, Singapore General Hospital, Singapore, Singapore; b Department of Molecular Pathology, Singapore General Hospital, Singapore, Singapore; c Genome Institute of Singapore, Agency for Science, Technology, and Research, Singapore, Singapore; d Yong Loo Lin School of Medicine, National University of Singapore, Singapore, Singapore; e Duke NUS Medical School, National University of Singapore, Singapore, Singapore; f Advanced Research Center for Computational Simulation, Chiang Mai University, Chiang Mai, Thailand; g Department of Infectious Diseases, Singapore General Hospital, Singapore, Singapore; h Department of Haematology, Singapore General Hospital, Singapore, Singapore; Johns Hopkins Hospital

**Keywords:** SARS-CoV-2, immunocompromised hosts, prolonged COVID-19

## Abstract

Immunocompromised hosts with prolonged severe acute respiratory syndrome coronavirus 2 (SARS-CoV-2) infections have been implicated in the emergence of highly mutated SARS-CoV-2 variants. Spike mutations are of particular concern because the spike protein is a key target for vaccines and therapeutics for SARS-CoV-2. Here, we report the emergence of spike mutations in two immunocompromised patients with persistent SARS-CoV-2 reverse transcription (RT)-PCR positivity (>90 days). Whole-genome sequence analysis of samples obtained before and after coronavirus disease 2019 (COVID-19) treatment demonstrated the development of partial therapeutic escape mutations and increased intrahost SARS-CoV-2 genome diversity over time. This case series thus adds to the accumulating evidence that immunocompromised hosts with persistent infections are important sources of SARS-CoV-2 genome diversity and, in particular, clinically important spike protein diversity.

**IMPORTANCE** The emergence of clinically important mutations described in this report highlights the need for sustained vigilance and containment measures when managing immunocompromised patients with persistent COVID-19. Even as jurisdictions across the globe start lifting pandemic control measures, immunocompromised patients with persistent COVID-19 constitute a unique group that requires close genomic monitoring and enhanced infection control measures, to ensure early detection and containment of mutations and variants of therapeutic and public health importance.

## OBSERVATION

Immunocompromised hosts with prolonged severe acute respiratory syndrome coronavirus 2 (SARS-CoV-2) infection have been implicated in the emergence of highly mutated SARS-CoV-2 variants (1–12). Unlike immunocompetent hosts, patients with compromised immunity from underlying medical conditions or immunosuppressive therapies have been reported to have high SARS-CoV-2 burdens for protracted periods (1, 3, 7, 9–11, 13). Accumulating evidence also suggests that these immunocompromised patients are at significantly increased risk of severe coronavirus disease 2019 (COVID-19), necessitating aggressive prophylaxis and therapy (14–18). The prolonged infection and consequent viral replication in immunocompromised hosts, combined with selection pressure exerted by therapies, constitute a rich reservoir from which therapeutic and immune escape mutations can emerge and accumulate. The intrahost evolution of the SAR-CoV-2 virus within this subgroup of patients might have played an important role in the emergence of the currently predominant B.1.1.529 (Omicron) variant and other variants, as evidenced by the multitude of mutations found in a single patient with advanced human immunodeficiency virus (HIV) disease (8, 13). The highly mutated Omicron variant, which partially evades Pfizer BNT162b2 vaccine and multiple therapeutic SARS-CoV-2 antibodies (19–23), has caused massive waves of infection globally since it was first described in November 2021 (24).

Therefore, immunocompromised patients with persistent COVID-19 constitute a unique patient group that requires close genomic monitoring and enhanced infection control measures, even as jurisdictions across the globe start embracing SARS-CoV-2 endemicity (25). The utility of near-real-time genomic monitoring of this high-risk patient group is 2-fold. First, the genomic data directly benefit individual patients by allowing the attending clinical team to tailor therapeutics based on any potential therapeutic escape mutations. Second, genomic surveillance allows emergent variants to be monitored to safeguard public health. Therefore, our institution has in place an on-site genomic surveillance program to enable near-real-time sequencing of SARS-CoV-2 samples. Here, we describe the emergence of multiple partial therapeutic escape spike mutations in two immunocompromised patients with persistent SARS-CoV-2 reverse transcription (RT)-PCR positivity, who were identified through our on-site genomic surveillance program.

**FIG 1:**
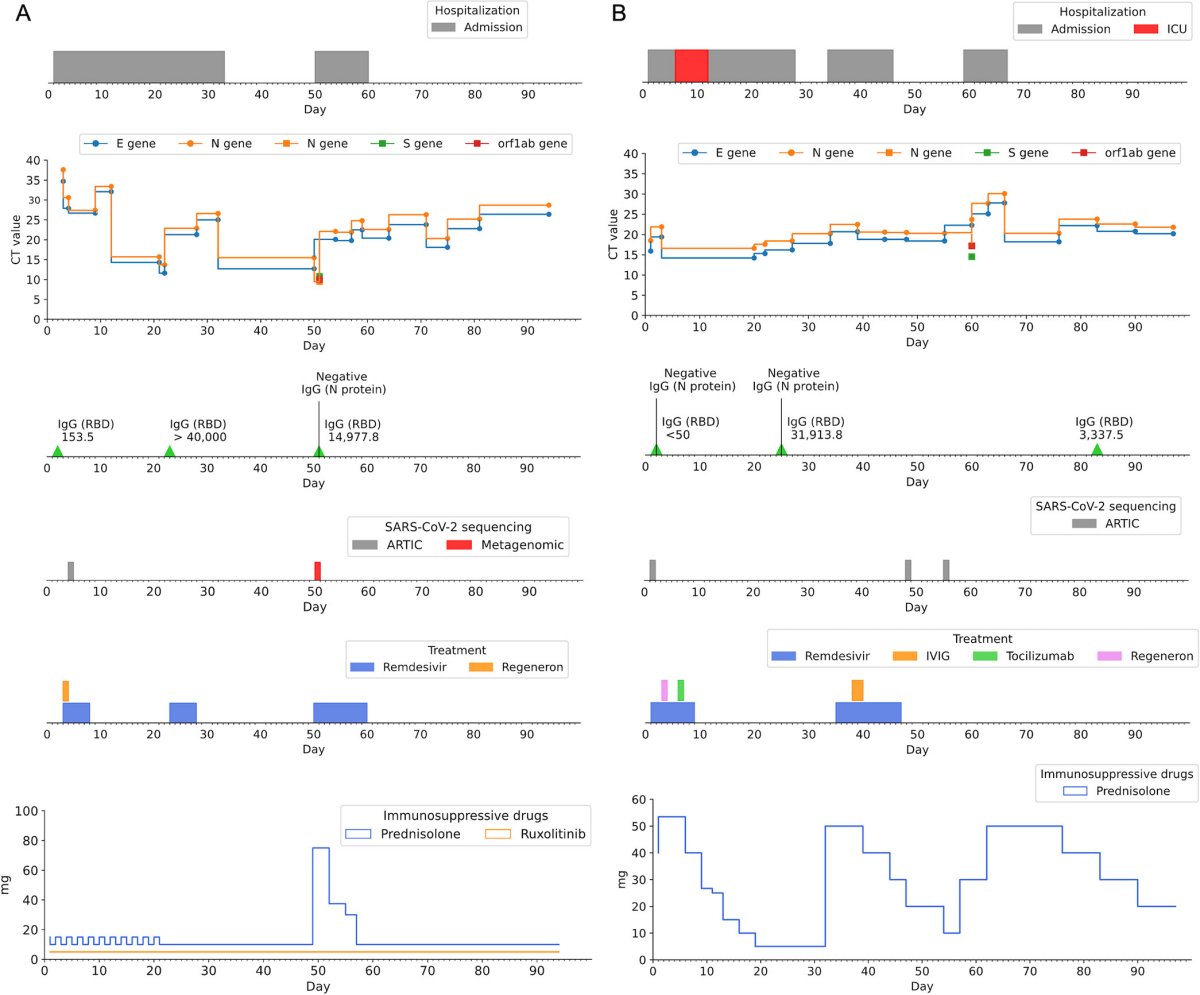
Summary and timeline of SARS-CoV-2 RT-PCR positivity, serology results, treatments, and key clinical events for patient A (A) and patient B (B). SARS-CoV-2 RT-PCR E gene and N gene *C_T_* values were obtained during routine diagnostic testing of nasopharyngeal swab samples using the Cepheid Xpert Xpress SARS-CoV-2 assay. The TaqPath Combo kit (Thermo Fisher Scientific) additionally detected S gene and ORF1ab targets in both patients’ samples (circles, Xpert Xpress SARS-CoV-2 assay; squares, TaqPath Combo kit). Serology tests for IgG against the SARS-CoV-2 RBD and N protein were performed during routine diagnostic testing using the chemiluminescent microparticle immunoassay (CMIA) on the Architect i2000SR system (Abbott Laboratories). Hydrocortisone (patient A, days 50 to 55) and dexamethasone (patient B, days 2 to 11) dosages were converted to prednisolone-equivalent doses for ease of visualization. Data used to generate this figure are available in Supplemental tables 1A and 1B.

Patient A is a fully COVID-19-vaccinated (two doses of Pfizer-BioNTech vaccine) 53-year-old man with a history of acute myeloid leukemia (AML-M2). He received an allogeneic stem cell transplant in January 2019. This was complicated by pulmonary chronic graft-versus-host disease (cGVHD). He had multiple opportunistic infections in the past, including Cytomegalovirus (CMV) retinitis, pulmonary aspergillosis and pulmonary nocardiosis. He was taking ruxolitinib (5mg daily) and prednisolone (15mg/10mg daily on alternate days) for the cGVHD when he caught Covid 19. His other significant comorbidities include visual impairment and end-stage renal failure requiring dialysis, secondary to diabetic nephropathy. Patient A was initially admitted on 15 November 2021 (day 1) for inpatient hemodialysis support when his primary caregiver tested positive for COVID-19. He was nursing a cough at admission and tested negative for SARS-CoV-2 by RT-PCR, as well as daily rapid antigen testing (SD Biosensor Standard Q COVID-19 antigen test) until day 3 of admission. On day 3, his chest X-ray showed right lower zone opacities, and he tested positive for SARS-CoV-2 by RT-PCR (Xpert Xpress SARS-CoV-2; Cepheid, USA). In view of his International Severe Acute Respiratory and Emerging Infections Consortium (ISARIC) 4C mortality score of 10 (26), he was promptly started on one dose of casirivimab (REGN10933) and imdevimab (REGN10987) cocktail (REGN-COV2), and a 5-day course of remdesivir, for SARS-CoV-2 pneumonia. He remained minimally symptomatic for COVID-19 but continued to test positive on SARS-CoV-2 RT-PCR. On day 21 of admission, a sudden drop in the RT-PCR cycle threshold (*C_T_*) value was observed (from an E gene *C_T_* value of 32.1 on day 12 to an E gene *C_T_* value of 14.3 on day 21), suggesting an acute increase in SARS-CoV-2 viral burden. He was started on a second 5-day course of remdesivir, and his prednisolone dose was reduced to 10 mg daily. He remained in clinically stable and was discharged on day 34. On day 50, he was re-admitted due to new-onset fever and worsening cough and breathlessness. SARS-CoV-2 RT-PCR testing on admission showed low *C_T_* values for the E gene (*C_T_* value of 14.3) and N gene (*C_T_* value of 15.7) targets, and the patient was started on a third course of remdesivir (10-day course) and monitored as an inpatient. His chest X-ray showed no new acute changes and resolution of the previously seen right lower zone opacities. His respiratory symptoms improved significantly on day 54, and he was discharged on day 61. He continued to test positive on RT-PCR to the time of writing, on day 94. Nasopharyngeal swab samples from day 4 (PA_D4) and day 50 (PA_D50) underwent whole-genome sequencing to investigate for possible immune and/or therapeutic escape mutations. Figure 1A summarizes the timeline, relevant investigations and treatments, and key clinical events.

Patient B is a fully COVID-19-vaccinated (two doses of Pfizer-BioNTech vaccine) 67-year-old woman who was recently diagnosed with splenic marginal zone lymphoma, status post splenectomy in March 2021. She has a longstanding history of Evans syndrome, which has been complicated by recurrent admissions for symptomatic autoimmune hemolytic anemia, for which she received multiple courses of steroids and intravenous rituximab therapy in 2018. Prior to her admission for COVID-19, she had been managed on prednisolone (40 mg daily) and was receiving acyclovir and trimethoprim-sulfamethoxazole prophylaxis. Patient B presented to the emergency department on 13 November 2021 (day 1) for worsening cough and breathlessness. Chest X-rays obtained at admission showed bilateral perihilar and left lower zone consolidation with air space opacity. The patient tested positive for SARS-CoV-2 by RT-PCR (Xpert Xpress SARS-CoV-2) and was started on remdesivir and dexamethasone on the same day. One dose of REGN-COV2 was given on day 3. On day 5, the patient clinically deteriorated with episodes of hypotension and hypoxemia. She was transferred to the high-dependency unit and was started on high-flow oxygen supplementation. She progressed to type 1 respiratory failure, and she was intubated and started on mechanical ventilation on the following day (day 6). Endotracheal aspirate cultures grew Aspergillus fumigatus complex, and the patient was initially treated with voriconazole for COVID-19-associated pulmonary aspergillosis. While she was in the intensive care unit, her hypotension responded to fluid resuscitation and noradrenaline, and her oxygenation improved progressively. She was given a dose of tocilizumab while she was in the intensive care unit. On day 11, she was extubated and weaned down to 2 L/min of oxygen via nasal cannula. During her 6-day stay in the intensive care unit, she was noted to have arrhythmia and deranged liver function test results; as a result, remdesivir treatment was stopped. The patient completed a 7-day course of remdesivir. She was transferred out of the intensive care unit to the general ward on day 12. Computed tomography scans of the chest performed on day 14 showed diffuse ground-glass changes in bilateral lungs and bilateral segmental and subsegmental pulmonary embolisms, for which the patient was started on a therapeutic regimen of enoxaparin. She persistently tested positive for SARS-CoV-2 throughout her admission, with low *C_T_* values for E gene (*C_T_* value of 16.2) and N gene (*C_T_* value of 18.4) targets on day 27. Her prednisolone dose was tapered progressively to 5 mg daily in an attempt to reduce the degree of immunosuppression, to facilitate clearance of SARS-CoV-2. She was discharged from inpatient service to telemedicine monitoring on day 28. She was subsequently readmitted on day 34 for symptomatic anemia secondary to a flare of autoimmune hemolytic anemia, likely contributed by the relatively low dose of prednisolone (5 mg) on which she had been maintained since discharge. Upon admission, she was started on high-dose prednisolone (50 mg). Her SARS-CoV-2 RT-PCR results continued to be positive (E gene *C_T_* value of 17.8 and N gene *C_T_* value of 20.2), and she was started on a second (10-day) course of remdesivir. Her prednisolone dose was again tapered to 10 mg to facilitate viral clearance, but she was admitted again for symptomatic anemia on day 59. She was discharged on day 67 with high-dose prednisolone (50 mg), which was subsequently tapered gradually to 20 mg at the time of writing. On day 97, her SARS-CoV-2 RT-PCR results remained positive. Nasopharyngeal swab samples from day 1 (PB_D1), day 48 (PB_D48), and day 55 (PB_D55) underwent whole-genome sequencing to investigate for possible immune and/or therapeutic escape mutations. Figure 1B summarizes the timeline, relevant investigations and treatments, and key clinical events.

Whole-genome sequencing of samples from patient A (PA_D4 and PA_D50) and patient B (PB_D1, PB_D48, and PB_D55) was first performed on a MinION MK1b system (Oxford Nanopore Technologies [ONT], Oxford, UK) in accordance with the ARTIC Network protocol v3 (27). The RAMPART protocol (https://github.com/artic-network/rampart) was used to monitor the depth of coverage for each sample and to construct a draft genome. Sample PA_D50 was also sequenced using a metagenomic approach to improve coverage of the SARS-CoV-2 genome. All samples sequenced with the ARTIC Network protocol (PA_D4, PB_D1, PB_D48, and PB_D55) achieved coverage of >97% and an average depth of >462×. For all samples, total nucleic acid extraction of the chemically inactivated remnant samples was performed on the Promega Maxwell RSC 48 instrument, using the Maxwell RSC viral total nucleic acid purification kit (Promega, USA). cDNA synthesis was performed using the LunaScript RT SuperMix (New England BioLabs, USA) according to the ARTIC Network protocol (27). For metagenomic sequencing of PA_D50, library preparation was performed using the SQK-LSK 109 ligation sequencing kit (ONT) according to the manufacturer’s instructions. PA_D50 was monoplex sequenced on an FLO-MIN106D flow cell (R9.4.1) for 72 h and achieved 100% coverage and an average depth of 173×.

Guppy v5.0.7 (in super accuracy mode) was used for the basecalling of all samples. Consensus sequences were generated using the ARTIC Network pipeline for SARS-CoV-2 v1.2.1 (https://github.com/artic-network/artic-ncov2019). Within the ARTIC Network pipeline, Medaka v1.5.0 and Longshot v0.4.1 were chosen for variant calling. For PA_D50, reads that were aligned to the host genome (GRCh38) using minimap2 v2.17 were filtered out, and the remaining reads were aligned to the SARS-CoV-2 reference genome (GenBank accession number NC_045512.2). Allele frequency information was extracted from the VCF files before the final filtering step, to preserve variants with alternate allele frequencies of less than 0.5. Lineages of all consensus sequences were determined based on PANGO v3.1.17. Lists of mutations were retrieved from CalmBelt, our in-house tool for analyzing SARS-CoV-2 consensus sequences (28). This study was approved by the SingHealth Centralized Institutional Review Board (protocol 2013/397/F).

Consensus sequences of initial samples from patients A and B (PA_D4 and PB_D1, respectively) indicated that both patients were infected with the B.1.617.2 (Delta) variant. No signature Omicron single-nucleotide variations (SNVs) were found to have emerged over the course of investigation of these two cases. Since both patients had received two doses of Pfizer-BioNTech vaccines more than 4 weeks prior to being diagnosed with COVID-19 and had detectable anti-receptor-binding domain (RBD) IgG titers, we looked for and found the putative vaccine escape mutation L452R (29, 30) in the initial samples from both patients (PA_D4 and PB_D1).

**FIG 2:**
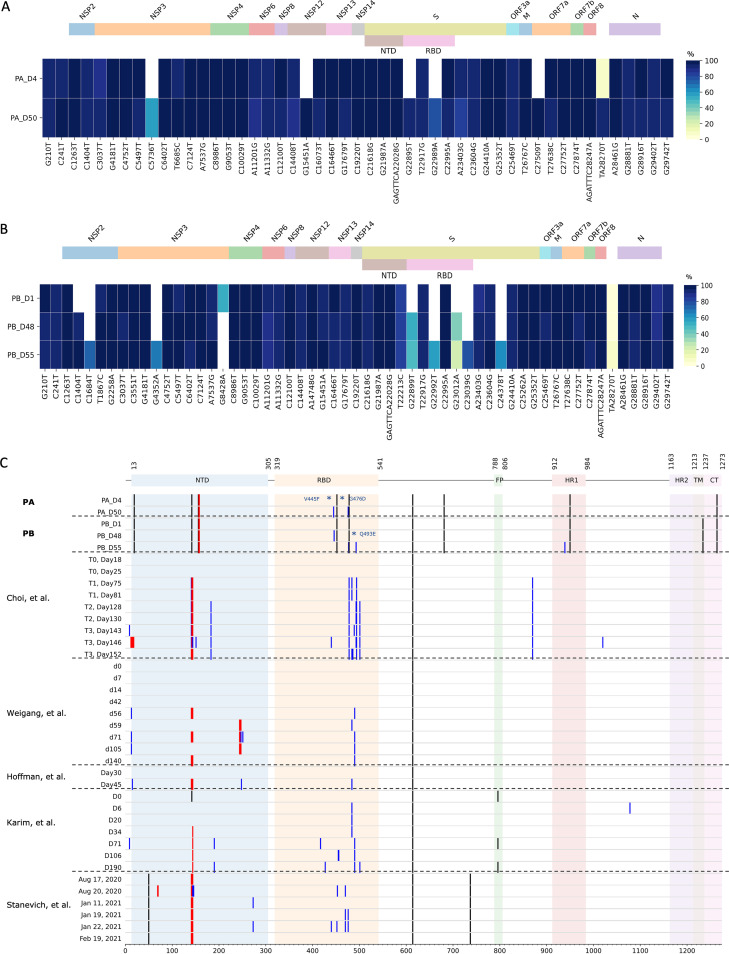
(A) Heatmap showing the position and frequency of viral genome variations in the day 4 (PA_D4) and day 50 (PA_D50) samples for patient A, compared to the reference genome (GenBank accession number NC_045512.2). The intensity of the heatmap illustrates the variant frequencies. Data used to generate this figure are available in Data Set S2 in the supplemental material. (B) Day 1 (PB_D1), day 48 (PB_D48), and day 55 (PB_D55) samples for patient B, compared to the reference genome. Data used to generate this figure are available in Data Set S2. (C) Schematic displaying locations of deletions and mutations of SARS-CoV-2 genomes based on consensus sequences. The samples from patient A (PA) and patient B (PB), as well as other published reports of persistent infections in immunocompromised hosts (2, 5, 9, 11, 13), are aligned to illustrate the accumulation of mutations in the RBD region. Red lines, deletions; black lines, substitutions; blue lines, emergent mutations not found in the patient’s initial samples; *, mutation not found in GISAID sequences uploaded from Singapore between 1 November 2021 and 28 February 2022. NTD, N-terminal domain; FP, fusion peptide; TM, transmembrane domain; CT, cytoplasmic tail.

For both patients, nasopharyngeal swab samples were sequenced before (PA_D4 and PB_D1) and after (PA_D50, PB_D48, and PB_D55) REGN-COV2 and remdesivir treatments. Compared to the initial sample for patient A (PA_D4), the day 50 sample (PA_D50) had five additional nonsynonymous mutations. The G5736T (A1006V) mutation occurred in the region encoding nonstructural protein 3 (NSP3), while the G27509T (T39I) mutation was found in the accessory protein open reading frame 7a (ORF7a) gene. The G15451A (G671S) mutation occurred in the NSP12 gene, which encodes a subunit of the RNA-dependent RNA polymerase (RdRp). Of note, two mutations (G22895T [V445F] and G22989A [G476D]) were found in the spike protein S1 subunit RBD. The mutations and associated allele frequencies of the samples from patient A are illustrated in Fig. 2A.

The day 48 (PB_D48) and day 55 (PB_D55) samples for patient B similarly showed accumulating mutations over time, compared to the initial sample (PB_D1). Similar to the findings for patient A, nonsynonymous mutations emerged in NSP3 (G4352A [E545K]) and the spike protein. Three spike protein mutations occurred in the S1 subunit RBD (G22899T [G466V], G22992T [S477I], and C23039G [Q493E]), and one was found in the S2 subunit heptapeptide repeat sequence 1 (HR1) region (C24378T [S939F]). One additional nonsynonymous mutation was observed in NSP2 (C1404T [P200L]). The mutations and associated allele frequencies of the samples from patient B are illustrated in Fig. 2B.

Whole-genome sequence comparison of samples from the patients in this case series showed the emergence and accumulation of mutations within a host over time. Despite differences in their clinical courses, both patients’ later samples demonstrated increased intrahost SARS-CoV-2 genome diversity. Of note, for both patients, more than one new major allele was found in the region encoding the S1 subunit RBD (Fig. 2A and B). This case series thus adds to the fast-accumulating evidence that immunocompromised hosts with persistent infections are important sources of SARS-CoV-2 genome diversity and, in particular, S gene diversity. Although patients in this case series were infected with the delta variant, our findings are in keeping with other recently reported cases of immunocompromised hosts with persistent SARS-CoV-2 infections caused by other variants (Fig. 2C).

The accumulation of mutations in the spike RBD is of particular concern because S protein is a key target for vaccines and therapeutics for SARS-CoV-2. Collectively, the patients in this study had new mutations at positions 445, 446, 476, 477, and 493 of the spike protein. Among these, substitutions at residue 477 were shown to be associated with broad resistance to monoclonal antibody panels (31). Substitutions at position 446 were reported to affect neutralization by both monoclonal antibodies and antibodies present in polyclonal sera (31–33), whereas substitutions at positions 445, 446, and 493 were reported to confer various degrees of monoclonal antibody neutralization resistance (34, 35). The day 50 sample for patient A had V445F and G476D substitutions, which were found to be partial escape mutations for REGN10987 (imdevimab) and REGN10933 (casirivimab), respectively, by deep mutational scanning (35). The day 55 sample for patient B similarly developed partial escape mutations G446V and Q493E for REGN10987 (imdevimab) and REGN10933 (casirivimab), respectively (35). These REGN-COV2 partial escape mutations in major alleles identified after REGN-COV2 therapy may be a consequence of SARS-CoV-2 intrahost evolution under selection pressure.

In this case series, both patients received multiple courses of remdesivir. Therefore, we examined the genome sequences for known remdesivir resistance mutations. No known or putative mutation conferring remdesivir resistance was found (36).

To understand whether patients A and B might have acquired a new SARS-CoV-2 infection in the period between the initial and subsequent samples, we examined the frequency of their new mutations in the GISAID database (https://www.gisaid.org). Among 4,255 GISAID sequences uploaded from Singapore between 1 November 2021 and 28 February 2022, no other sequence harbors the C5736T, G22895T, or G22989A mutations found in the day 50 sample for patient A. Similarly, the G4352A and C23039G mutations in the samples from patient B were not found in the same data set. Among >3 million GISAID sequences uploaded globally between 1 November 2021 and 28 February 2022, the occurrence of these mutations ranged from 0.001% to 0.017%. The data suggest that patients A and B were unlikely to have acquired a new infection with a separate SARS-CoV-2 strain with these mutations.

There are several limitations to this study. Although prolonged SARS-CoV-2 RT-PCR positivity was established, viral culture of the samples was not performed to confirm virus viability or infectivity. Furthermore, the application of whole-genome analysis provided a static glimpse of the dynamic viral subpopulations in the hosts. ONT sequencing was used in our setting within a hospital diagnostic environment, to reduce costs and turnaround time. Although it has been established that SARS-CoV-2 ONT sequencing has high accuracy, compared to short-read sequencing, ONT sequencing generally achieves lower sequencing depth, compared to short-read sequencing (37).

Nonetheless, this case series adds to the literature two cases of prolonged SARS-CoV-2 infection, with the emergence of clinically important mutations, in immunocompromised hosts. Relatively low-cost and simple on-site SARS-CoV-2 whole-genome sequence analysis identified putative vaccine escape mutations in the initial samples, as well as the emergence of partial therapeutic escape mutations in the subsequent samples. This emergence and accumulation of escape mutations, as well as the prolonged course of infection, put individual vulnerable hosts at risk of therapeutic failures and severe infections. Furthermore, the emergence and spread of such variants harboring vaccine and therapeutic escape mutations have important public health and infection control implications. Although some jurisdictions have progressively lifted pandemic restrictions, tertiary centers caring for immunocompromised patients need to exercise continued vigilance and containment measures against such emergent variants. Routine and near-real-time SARS-CoV-2 genomic surveillance in this subgroup is necessary for early detection and containment of mutations and variants of therapeutic and public health importance.

### Data availability.

Sequence data were submitted to GenBank with the following accession numbers: OM881791 (PB_D55), OM881787 (PB_D48), OM881786 (PA_D50), OM877509 (PB_D1), and OM877508 (PA_D4).
